# Enhance VR: A Multisensory Approach to Cognitive Training and Monitoring

**DOI:** 10.3389/fdgth.2022.916052

**Published:** 2022-06-03

**Authors:** Victòria Brugada-Ramentol, Amir Bozorgzadeh, Hossein Jalali

**Affiliations:** Virtuleap, Lisbon, Portugal

**Keywords:** virtual reality, cognitive training, cognitive monitoring, immersion, multisensory integration, presence

## Abstract

Cognitive training systems aim to improve specific domains or global cognition by engaging users in cognitively demanding tasks. While screen-based applications can improve performance in the trained cognitive abilities, they are often criticized for their poor transferability to activities of daily living. These systems, however, exclude the user's body and motor skills, which invariably serves to restrict the user experience. Immersive Virtual Reality (IVR) systems, in contrast, present the user with body-related information, such as proprioceptive and visuomotor information, allowing for an immersive and embodied experience of the environment. This feature renders VR a very appealing tool for cognitive training and neurorehabilitation applications. We present Enhance VR, an IVR-based cognitive training and monitoring application that offers short daily cognitive workouts. The games are designed to train and monitor specific cognitive domains such as memory, task flexibility, information processing, orientation, attention, problem-solving, and motor control. The aim is to test whether cognitively demanding tasks, presented in an IVR setting, provide a naturalistic system to train and monitor cognitive capabilities.

## 1. Introduction

**Figure 1:**
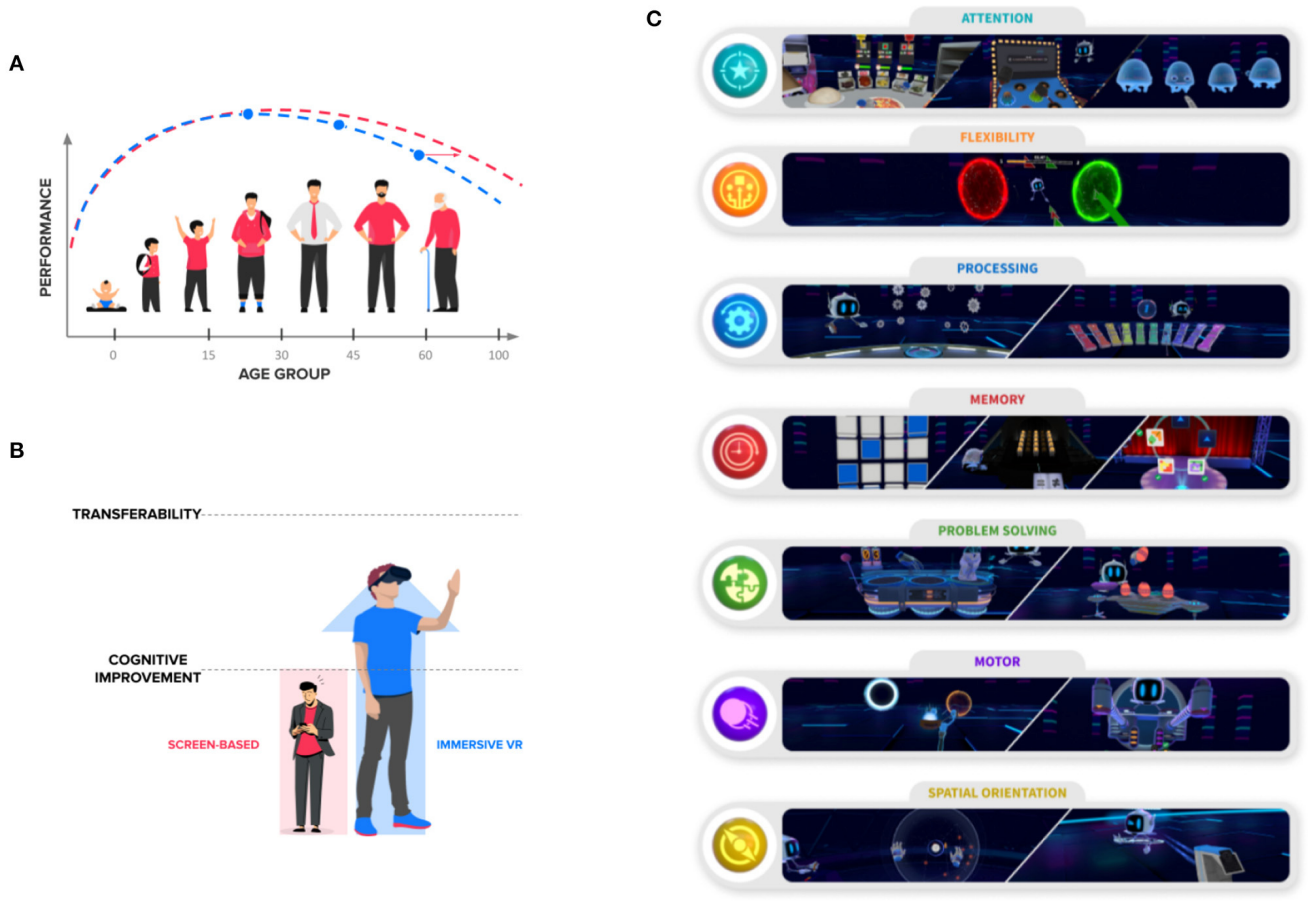
Enhance VR offers a library of cognitive exercises in virtual reality. **(A)** Cognitive abilities change across the lifespan of an individual. Cognitive training solutions, together with physical exercise and a healthy balanced diet, have shown potential improvement in the cognitive abilities of elderly. **(B)** Screen-based cognitive training solutions have shown potential improvement in the trained cognitive abilities, but the effects on global cognitive functioning and activities of daily living are still to be determined. Virtual reality systems present the user with an embodied experience of cognitive exercises, which can potentially increase the possibility of transfer of benefits. **(C)** The Enhance VR exercises span across seven cognitive categories (and their respective subcategories). Namely, attention, cognitive flexibility, information processing, memory, problem-solving, motor control, and spatial orientation. The latter being inherent to IVR environments.

Cognitive decline, the gradual deterioration of cognitive abilities and functioning, progresses in parallel with age and becomes more pronounced in populations at risk of neurodegenerative diseases (Figure 1A). The deterioration of cognitive abilities has a direct influence on the execution of activities of daily living (ADLs) and negatively affects autonomy and wellbeing. As life expectancy continues to increase, the number of the senior population and, consequently, the prevalence of cognitive impairment is expected to increase (WHO Europe, n.d.). A combination of healthy diet, moderate exercise, and cognitive stimulation have been proposed as strategies to attempt to slow down the progression of cognitive decline (1–3). Among these, cognitive training systems aim to maintain an effective cognitive function through the structured practice of specific cognitive domains. Furthermore, when presented in a computerized form, the exercise difficulty can be adapted to the performance of the individual, which presents an advantage over pen-and-paper formats (4).

Thus, computerized cognitive training (CCT) could potentially act as a non-pharmacological intervention to maintain cognitive functioning across the lifespan of healthy and cognitively impaired individuals. CCT has been shown to reduce cognitive decline in healthy aging (5), but with no clear effectiveness in delaying difficulties in instrumental ADLs. CCT programs have shown an improvement in trained cognitive abilities (i.e., memory, reasoning, or speed-of-processing) (6), which was maintained for 5 (7) or even 10 years (8). A meta-analysis found a small and significant effect on cognitive function in healthy older adults (9). Overall, CCT has shown promising, but heterogeneous results in global cognitive functioning (10). However, screen-based CCT solutions have failed to show transfer to untrained cognitive categories and tasks and iADLs (6, 11), thus failing to show transfer of benefits. Furthermore, computer-based interventions showed improvements in cognition and non-cognitive measures (e.g., mood), but failed to improve ADLs in people with dementia (12). A 12-week program showed improved capacities in early-stage Alzheimer's disease (AD), without positive effects on ADLs (13).

The lack of ecological validity in screen-based CCT could, in part, explain why these systems do not result in a transfer of benefits to ADLs and general cognition. While screen-based systems fail to provide body-related sensory information (e.g., proprioception), virtual reality (VR) environments provide it innately through multisensory embodied experiences (14). VR environments yield ecologically valid environment scenarios with precise control over the experimental variables (15, 16). VR has shown to be potentially relevant for cognitive training in the elderly population, as a result of many different features, such as the flexibility of the environments and the possibility to gather rich data, as a result of increased immersion (17). Thus, cognitive training in VR can potentially improve cognitive functioning in healthy and at-risk older adults and promote the transfer of benefits (Figure 1B).

## 2. Virtual Reality Environments as a Cognitive Training and Monitoring Tool

### 2.1. Advantages of Using VR Environments

VR scenarios offer many advantages over screen-based cognitive training methodologies. Traditional methodologies have to trade external validity (i.e., how the task accurately represents the measure in real life scenarios) and the internal validity of conducting the tasks in a controlled laboratory setting with controlled variables (18). VR, on the other hand, offer realistic scenarios with a high degree of control over desirable experimental variables (15), such as the stimuli, the presence of distractors, among others. Additional advantages of VR systems include the possibility to create situations that are adapted to the individuals needs or that are impossible to recreate in real life scenarios and collect precise measurements of physical movements, while sourcing large amounts of behavioral data (18).

Moreover, immersive VR (IVR) systems that are displayed through a head-mounted display with embedded head-tracking, which update the environment according to the movement of the participant, allow for a naturalistic interaction with the environment. In these scenarios, the behavior of the environment matches the expected physical and motor consequences of the action of the participant. This is also referred to as the plausibility illusion. Furthermore, IVR environments engage the sensorimotor system enhancing the illusion of embodiment over a virtual body or body part (19, 20). By providing immersive experiences of the scenarios, virtual environments provide the feeling that the virtual world is the real world and provide the feeling of actually “being” in the virtual environment (i.e., placement illusion). The feeling of presence in virtual environment has shown to increase motivation for learning and attention to the task (21) and it is dependent both on hardware and software (22). These three illusions contribute to the immersive experience of the environment (23).

Sensorimotor contingencies are, thus, important for the immersion. VR systems are particularly interesting for this matter, as they allow for the integration of proprioceptive, visual, and motor information (14). Ultimately, VR scenarios provide a high degree of realism that elicit naturalistic behaviors from participants (24). Together with the sense of presence and immersion in the virtual environment, the naturalistic interaction with the environment provides a higher degree of ecological validity than current screen-based or pen-and-paper solutions. As a result, VR systems become interesting tools for training and rehabilitation of cognitive functions in distinct populations (25).

### 2.2. Cognitive Training and Rehabilitation in VR

VR enables a stronger sensory immersion that promotes higher cognitive processing and learning and has found positive impacts on learning outcomes when analyzing the effects of VR-based games and simulations (21, 26, 27). In addition, VR enhances the learning and the application of information compared to screen-based systems (28). VR is increasingly being used in the field of cognitive rehabilitation, such as rehabilitation of post-stroke patients (25) and indicated potential applications for Parkinson's disease (29). Training in wayfinding paradigms in VR has shown improvement in the untrained memory category (30). Lastly, VR has proven itself useful in the study of postural instability in AD patients (31).

Exercises in semi-immersive VR representing a virtual supermarket mixed with physical activity (such as using a treadmill) has been proposed as an alternative that could ultimately translate to improvements in daily life activities (32). Evidence using a VR cognitive training system in patients with mild cognitive impairment (MCI), a prodromal state to AD, shows moderate effects in global cognitive functioning (33). Stroke rehabilitation using an ecological valid simulating ADLs scenario can have a positive impact in post-stroke individuals, showing larger improvements than conventional methods (34). Individuals with MCI and dementia have reported a preference for VR formats over the pen-and-paper versions, thus, suggesting the potential to increase engagement and adherence to non-pharmacological interventions (35). A VR cognitive training system, representing ADLs showed positive improvement in executing function and visual memory in elderly, and general cognitive (36). A program designed to train memory, attention, and executive function resulted in improvements in the healthy and MCI individuals (37).

The advantages of IVR are not only restricted to cognitive training, but also to provide immersive scenarios for cognitive assessment (38). As a result of the naturalistic interaction and the realistic scenarios, VR cognitive assessment offers the opportunity to accurately measure the cognitive performance of the individual.

## 3. Enhance VR, a Cognitive Training and Monitoring Tool in Virtual Reality

Enhance VR is a commercially available app consisting of a library of cognitive exercises (hereafter, games) developed by Virtuleap (Virtuleap, United States, virtuleap.com). The Enhance VR system takes advantage of the multisensory experience of immersive VR environments to train and monitor specific cognitive categories (i.e., memory, attention, task flexibility, information processing, orientation, and problem-solving) and motor control (Figure 1C).

The Enhance VR app is designed to be played regularly in the form of short workouts. Each workout is composed of three randomly chosen games. The mechanics of each game is motivated by the mechanics of validated neuropsychological principles. Every game stems from the collaboration between scientists and game designers to ensure that the mechanics of each test are maintained, while the experience is a gamified and engaging representation of the neuropsychological validated tests (Table 1). Every game starts with a benchmark session that aims to find the current level of the participant's performance. From there on, every session starts at the level where the user left off. The difficulty of the Enhance VR app is game dependent and controlled by adjusting the relevant parameters for each exercise. The Enhance VR is compatible with a wide range of commercially available headsets (https://virtuleap.com/download/).

**Table 1:** Enhance VR game descriptions.

**Game name**	**Category**	**Neuropsychological test**	**Game description**
Pizza builder	Attention	Dual-task paradigms (39)	Assemble multiple pizzas according to continuously incoming orders
Whack-a-mole	Attention	Psychomotor vigilance test (40)	Hit the moles as they pop up from the arcade.
Shuffled	Attention	Moving boxes task (41)	Track a single jellyfish and ignore the rest as they move around.
React	Flexibility	Wisconsin card sorting test (42) and Stroop task (43)	Throw objects into portals according to their shape and color.
Assembly	Information processing	Trail making test (44)	Select, one by one, gears from a group in ascending size.
Harmonize	Information processing	Paced visual serial addition task (45)	Add up the two last digits displayed on a screen.
Memory Wall	Memory	Visual patterns test (46)	Memorize and reproduce a pattern of cubes.
Maestro	Memory	n-back task (47)	Memorize light patterns and report when the patterns repeat.
Magic deck	Memory	Paired-associate learning (48)	Memorize the positions of cards containing abstract patterns.
Stacker	Problem-solving	Towers of Hanoi (49)	Reconstruct a statue moving only one piece at a time.
Odd Egg	Problem-solving	Odd one out (50)	Find the element that stands out from the group.
Slinger	Motor	Target test (51)	Hit all the targets before they disappear.
Balance	Motor & Attention	Motor-cognitive dual-task paradigm (52)	Maintain the required amount of spherical objects onto two plates.
Orbital	Spatial orientation	Mental rotation task (53)	Direct a spaceship to its destiny by finding the correct path.
Hide and Seek	Spatial orientation	Selective auditory attention test (54)	Localize the origin of an auditory cue while ignoring the distractor sounds.

*The Enhance VR library is composed of 15 games (April 2022). The games are designed to train or monitor one of the seven Enhance VR categories. The mechanics of each game is designed based on validated neuropsychological tests*.

Individual progression is tracked by the Enhance VR Performance Index (EPI), calculated as an aggregate of weighted performance across all cognitive categories in addition to scores for each game, the main cognitive categories, and subcategories scores. The app collects data on self-reported mood and sleeping hours at the start of each workout. Most importantly, there are also game-play patterns that can be obtained from the collection of behavioral data obtained by game-specific events, ultimately allowing the Enhance VR system to calculate reaction times, accuracy, and other behavioral variables.

## 4. Discussion

With the extension of life expectancy and the aging of the population, the numbers of the elderly population affected with cognitive impairment and loss of autonomy are expected to increase. The number of older adults affected by AD is projected to reach 1.6 billion globally by 2050 (55). It is, therefore, crucial to develop and implement strategies that extend the autonomy and independence of individuals at risk of cognitive decline. Pharmacological interventions have yet to be proven fully useful in helping arrest cognitive and functional decline in those suffering from neurodegenerative diseases. Among potential non-pharmacological interventions, cognitive training presents an opportunity to extend the individual's autonomy and independence (2, 56).

Exposure to a multi-domain active video game itself has been shown to result in a transfer of benefits between tasks (57), which was maintained up to a six-month follow-up period. Thus, gaming environments are a promising tool for the transfer of benefits (58). Training in game-like IVR scenarios has been proposed as a means to increase engagement and motivation in neurorehabilitation (59). Furthermore, immersive VR scenarios offer an ecologically valid environment that can potentially improve global cognitive functions and have positive effects on untrained cognitive abilities. The Enhance VR app takes advantage of the immersive and ecological validity of VR environments to provide a structured and controlled setting for cognitive training. The Enhance VR app can be considered a cognitive training system that offers mentally challenging exercises that aim to train specific cognitive skills presented in a structured manner and extracts behavioral measures of cognitive fitness (60). The Enhance VR app has the potential to enable strong presence and high placement and plausibility illusion, which increases the ecological validity of its environment (61).

To this day, few studies have used IVR environments for cognitive training in healthy elderly (17, 62) or MCI (63) populations. Recent studies point toward positive attitudes displayed by the elderly population in the use of VR environments (64). Further, studies have shown promising results regarding the acceptability and usability in elderly individuals with subjective cognitive impairment and MCI (65) and patients with mild dementia (66), overall suggesting that structured and controlled environments for cognitive training pose a promising tool for elderly at risk of cognitive decline. We therefore propose that exposure to cognitively demanding, physically engaging, and sensory-rich immersive and gamified exercises, such as the ones offered through Enhance VR games, can potentially increase the transfer of benefits to iADLs and global cognitive functioning in populations at risk of cognitive decline.

IVR systems present a unique opportunity to collect potentially ecologically valid data (15) and cognitive assessment systems have shown to be useful in the assessment of cognitive functions (38). Therefore, the prolonged and regular engagement with the Enhance VR app enables the collection of longitudinal data that represents a naturalistic scenario. Thus, the Enhance VR app could also act as a cognitive monitoring tool. VR adaptations of the test have proven useful in the assessment of memory functions (67).

The design of each individual Enhance VR game can be compared to other workflows proposed for the design of cognitive assessment exercises (68). Whenever the need for a specific cognitive category is identified, different classical paradigms are selected and evaluated to maximize the training abilities in an IVR environment. Once the candidate paradigm has been identified, the extensive literature review identifies task requirements. Namely, (1) the mechanics of the paradigm, (2) the actions that are required from the participant, (3) the category and type of stimuli, (4) the outcome measures that are expected from the task that are critical to evaluate the cognitive ability, and (5) identify the parameters that can be modified to increase the difficulty of the task. Further evaluation of the Enhance VR app will require confirmation of the face and content validity of the tests since the inclusion of a strong motor component may add confounding variables despite careful co-design between scientific and development teams.

Additionally, VR scenarios offers the opportunity to assess cognitive function in realistic daily-life scenarios with increased ecological validity (38). For instance, VR paradigms enable testing of navigational skills in a supermarket and collecting ecologically relevant data, without exposing the participant to the dangers of navigating these actual scenarios (i.e., experimentally well-controlled) (68). The mechanics of the Enhance VR games derive from neuropsychological principles, such as the n-back task (47) or the Stroop task (43). We propose that the VR versions of the cognitive assessment tests could provide a naturalistic approach toward the measurement of cognitive capabilities since IVR promotes multisensory body-related information (69).

An additional advantage of IVR systems is the ability to collect large and varied behavioral datasets (17). As the participants engage with the Enhance VR games, the system not only collects the performance of the participants, but also a large number of game-related variables. Some of these variables include motor outputs (e.g., movement coordinates or hand preferences), and reaction times, among others. These data points could provide, when combined with supervised machine learning algorithms, novel non-invasive digital biomarkers of cognitive status by collecting characteristics from digital health technologies that could be used to monitor biological or pathogenic processes and assess responses to pharmacological interventions (70). The app thus affords, for an experimenter-independent monitoring of cognitive function, while allowing the researchers and clinical staff to access this data. The Enhance VR app is compliant with GDPR and HIPAA regulations to ensure that it can meet the criteria of data privacy (61).

The use of VR environments for cognitive training and monitoring (or assessment) presents some concerns that need to be addressed as their use is becoming more widespread (61, 68). Among these, the need to minimize adverse effects is of utmost importance. For instance, cybersickness can negatively affect the cognitive performance of the individuals (71). Efforts to minimize cybersickness can be implemented both in the hardware and software (61). First, the Enhance VR app takes advantage of the technical aspects of the hardware, supporting state of the art off-the-shelf headsets. The Enhance VR app is available for headsets with six degrees of freedom (6DoF), which provide a seemingly naturalistic interaction with the environment by the use of controllers (72). Additionally, the Enhance VR app provides high-quality software that can potentially help avoid cybersickness (61). Finally, the participant is not required to move within the virtual environment, which further helps reduce the potential for cybersickness. Future studies will assess the presence of adverse effects in VR environments (73).

The cost of the hardware has been reduced significantly in the last years and it is becoming more readily available for research and clinical purposes (23). However, the development of high quality immersive environments is time consuming and expensive (68). Thus, it is important to foster collaboration between enterprizes with the resources to develop the cognitive training software and research institutions with the means to validate these systems. The Enhance VR system is readily available for research purposes. Furthermore, it is accompanied by additional features that make it an interesting tool, such as a data dashboard that allows the monitoring of users within an organization; a remote control feature that allows for telehealth interventions; and a customizable survey system integrated within the Enhance VR system.

## 5. Conclusions

As the senior population continues to grow, it becomes crucial to implement strategies to maintain their autonomy and independence. Among the proposed strategies, cognitive training solutions provide a structured system to train multiple cognitive abilities in a scenario with an adaptive difficulty. While computerized cognitive training solutions have shown positive results, screen-based systems lack naturalistic interaction and show a limited transfer of benefits to activities of daily living and global cognitive functioning.

IVR systems, on the other hand, present the user with a multisensory environment, resulting in an immersive and embodied experience of the virtual scenario. By taking advantage of this feature, the Enhance VR app aims to train and monitor cognitive functions using cognitively demanding immersive games. The presentation of seemingly naturalistic environments could provide an advantage over screen-based systems, whereby Enhance VR can become a validated tool for cognitive monitoring and training.

## Data Availability Statement

The original contributions presented in the study are included in the article/supplementary material, further inquiries can be directed to the corresponding author/s.

## Author Contributions

All authors contributed to the manuscript writing, revision, and approval of the submitted version.

## Conflict of Interest

AB and HJ are Chief Executive Officer and Chief Technical Officer, respectively, and founders at Virtuleap. VB-R was employed as Neuroscientist at Virtuleap.

## Publisher's Note

All claims expressed in this article are solely those of the authors and do not necessarily represent those of their affiliated organizations, or those of the publisher, the editors and the reviewers. Any product that may be evaluated in this article, or claim that may be made by its manufacturer, is not guaranteed or endorsed by the publisher.
